# A multi-cognitive PCB defect detection model integrating Mamba

**DOI:** 10.1038/s41598-026-49734-2

**Published:** 2026-04-25

**Authors:** Lingxiao Jin, Yuqin Feng, Hao Yang, Shuxian Liu

**Affiliations:** School of Computer Science and Technology, Xinjiang University, Urumqi, 830017 China

**Keywords:** Printed circuit board, Defect detection, Multi-receptive field feature fusion, Multi-cognitive visual augmentation, Lightweight design, Engineering, Mathematics and computing

## Abstract

Printed Circuit Boards (PCBs) pose significant challenges for defect detection due to their complex textures, small defect targets, and subtle inter-class similarities. Traditional inspection methods are limited in robustness, while many deep learning-based detectors struggle with insufficient tiny-target feature extraction, low feature utilization, and high model complexity. To address these limitations, this study introduces PCB-MMF, a multi-cognitive hybrid framework integrating the Mamba state space model. The proposed MM-NET backbone combines CNN-based local feature extraction with Mamba-based global modeling, augmented by a Three-Stage Multi-Receptive Module (TSMR) to fuse global and multi-scale features while mitigating redundancy. A Multi-Cognitive Visual Augmentation Module (MC-VAM) enhances attention to critical regions and preserves shallow features through residual connections, while a Lightweight Group-Shared Detection Head (LGSD) applies parameter sharing to reduce computational cost without compromising accuracy. Experimental results on HRIPCB, DeepPCB, and DsPCBSD+ datasets demonstrate that PCB-MMF achieves mAP50 scores of 93.43%, 98.68%, and 85.39%, respectively. Furthermore, additional generalization experiments on the NEU-DET dataset achieve an mAP50 of 76.69%, confirming the robust performance of PCB-MMF across different industrial scenarios. Compared with the YOLO11 model, PCB-MMF reduces the number of parameters by 8.9% (from 2.58 to 2.35M) and the computational load (FLOPs) by 12.70%. Relative to the Mamba-YOLO model, PCB-MMF reduces the number of parameters by 58.48% and the computational load (FLOPs) by 55.28%. These findings confirm that PCB-MMF offers a favorable balance between accuracy and efficiency, providing a promising solution for lightweight, high-precision PCB defect detection in industrial applications.

## Introduction

With the rapid development of information technology, the popularity and integration of electronic products continue to increase. As a core component of electronic systems^1^, printed circuit boards (PCBs) face higher requirements for performance stability and anti-interference capability. However, due to the miniaturization of components, manufacturing complexity, and environmental factors (such as mechanical friction, electrostatic interference, and chemical corrosion), defects including missing holes, mouse bites, open circuits, and short circuits frequently occur during PCB fabrication. These defects directly threaten the reliability and safety of electronic devices^2^, highlighting the urgent need for precise defect detection to ensure quality control.

Traditional PCB defect detection methods, such as manual inspection, electrical testing, and infrared imaging, generally suffer from low efficiency, high risk of damage, and limited defect coverage. Automated Optical Inspection (AOI), as a non-contact detection technology, has therefore developed rapidly. Its core algorithms can be categorized into two groups^3–5^: traditional machine vision-based methods and deep learning-based approaches.

For automatic PCB defect detection, researchers have proposed a variety of machine vision-based methods. Wan et al.^6^ and Virasova et al.^7^ adopted Hough transform and Canny edge detection to extract image features and accurately detect missing holes by template matching. Baygin et al.^8^ also combined Hough transform and Canny operator for precise defect localization. Wong et al.^9^ enhanced PCB images using terahertz continuous wave imaging, followed by sync convolution and blind deconvolution, improving image enhancement quality. Liu et al.^10^ employed image smoothing, contrast enhancement, and sharpening, together with a combination of digital morphology and threshold segmentation, to recognize defects. Ye et al.^11^ extracted connected domains and geometric features of solder pads via morphological preprocessing and applied Principal Component Analysis (PCA) to improve efficiency and robustness. Chang et al.^12^ optimized particle swarm optimization (PSO) for efficient image segmentation and further combined FLANN (Fast Library for Approximate Nearest Neighbors) and SURF (Speeded-Up Robust Features) to achieve robust feature point matching. Nevertheless, traditional machine vision approaches are strongly affected by environmental lighting, imaging conditions, and image resolution, resulting in limited robustness and poor generalization, which can easily cause false detection and missed detection, often requiring manual post-correction.

In contrast, deep learning-based PCB defect detection methods demonstrate superior feature extraction capability and stronger generalization. By learning from large-scale defect samples, these methods significantly improve detection accuracy. Ding et al.^13^ proposed TDD-net based on Faster R-CNN, which utilized k-means clustering to obtain more reasonable anchors and multilevel feature mappings, enabling accurate PCB defect detection. Ancha et al.^14^ developed CSPGhost-YOLO, which integrated GhostC3 modules and sophisticated dataset augmentation to alleviate class imbalance and ensure high-speed real-time inference. Lan et al.^15^ improved YOLOv3 by adopting k-means++ clustering for anchor refinement, batch normalization for accelerated inference, and GIoU loss for enhanced detection. Inspired by these advanced methodologies, particularly the robust augmentation strategies demonstrated in^14^, we incorporate a comprehensive dataset augmentation pipeline to ensure the structural stability and generalization of our proposed PCB-MMF model. Xiao et al.^16^ incorporated coordinate attention into YOLOv7-tiny to enhance feature extraction, thereby improving detection accuracy. Tang et al.^17^ developed PCB-YOLO, which integrates k-means++ clustering, Swin Transformer blocks, small-object prediction layers, EIoU loss, and depthwise separable convolution to improve detection efficiency and real-time performance. Furthermore, Ancha et al.^18^ proposed TRSBi-YOLO, which leverages C3TR modules and a Bidirectional Feature Pyramid Network (BiFPN) to achieve high-precision feature fusion for real-time edge deployment. Zhu et al.^19^ proposed SRN-Net, a small-object detection framework tailored for PCB defects, which incorporates a separated global context attention (SGC) mechanism into the backbone network to enhance small-object awareness. Wu et al.^20^ designed EEM-Net with an efficient dual-attention mechanism to capture global context while maintaining low computational cost. Liu et al.^21^ introduced a Transformer-based detection model leveraging multi-scale features to improve the detection of tiny defects. Li et al.^22^ proposed a selective feature enhancement module that focuses on foreground information and suppresses irrelevant background, thereby better exploiting defect features. More recently, Wang et al.^23^ developed HSA-RTDETR, a PCB defect detection method based on RT-DETR and hierarchical scale-aware attention (HSA), which achieves efficient and accurate detection through multi-level improvements.

Despite the improvements achieved by the aforementioned methods, industrial PCB defect detection still faces a critical architectural dilemma. Models must simultaneously achieve global contextual perception to understand complex wiring backgrounds and retain hyper-local sensitivity to detect microscopic flaws, all while operating under strict computational constraints. Specifically, several issues remain to be addressed:

1. The CNN-Transformer Architecture Dilemma: Restricted by the small receptive field of CNN architectures and the insufficient local fine-grained feature extraction capability of Transformer architectures, models exhibit inadequate tiny-target feature extraction, low feature information utilization, and high complexity due to redundant information participating in computations.
2. Loss of Shallow Features in Deep Extraction: In the deep feature extraction stage, conventional attention mechanism structures such as C2PSA limit the model’s attention to complex scenes, and shallow features (which contain rich detailed information crucial for PCB inspection) have insufficient participation, leading to the submergence of tiny defects and poor small-target detection performance.
3. Edge Deployment Bottlenecks: In the bounding box delineation stage, the large number of model parameters results in excessively high computational load, which fundamentally fails to meet the low-latency deployment requirements of edge devices.

To solve these issues, this study selects the lightweight YOLO11n (Nano scale) as the baseline model and proposes the PCB-MMF multi-cognitive defect detection model integrating Mamba. By leveraging Mamba’s inherent linear-time sequence modeling capability, our approach perfectly bridges the CNN-Transformer gap—offering global receptive fields without the quadratic computational bottleneck. Specifically, the main contributions of this work are summarized as follows:

1. A MM-NET (Multi-Modal Fusion Network) feature extraction network with a hybrid architecture combining CNN and Mamba is designed. The network is divided into a CNN layer (for local feature extraction) and a Mamba layer (for global feature relationship construction). The Mamba layer innovatively embeds the TSMR (Three-Stage Multi-Receptive Module, TSMR) module, which combines global and local multi-fine-grained feature information to enhance the model’s ability to recognize tiny PCB defect features. It also applies identity mapping to partial edge information to reduce feature redundancy in high-dimensional space, thereby lowering model complexity.
2. A MC-VAM (Multi-Cognitive Visual Augmentation Module, MC-VAM) module is designed and embedded in the deep feature extraction stage. By adaptively weighting and fusing multi-scale feature information, this module improves the model’s attention to complex regions. Deep residual connections are used to enhance the utilization of shallow feature information, preventing small targets from being submerged in deep networks and avoiding feature information loss.
3. A LGSD (Lightweight Group-Shared Detection Head, LGSD) detection head is proposed. Through parameter sharing via Group Normalization (GN) and re-weighting using an attention mechanism, the LGSD significantly reduces the number of model parameters while maintaining excellent detection performance.

## Related work

### Real-time object detectors

The YOLO series has continually evolved to balance detection accuracy with computational efficiency. YOLOv5^24^ adopts the CSPDarknet53 backbone network, which reduces computational redundancy through cross-stage partial connections and improves feature transfer efficiency. The core innovations of YOLOv8^25^ include an Anchor-Free design and a decoupled head, utilizing the C2f module to facilitate lightweight and efficient feature fusion. Addressing post-processing efficiency, YOLOv10^26^ introduces a consistent dual assignment strategy for NMS-free training, which eliminates the need for Non-Maximum Suppression during inference and significantly reduces end-to-end latency.Recent iterations further refine spatial awareness and computational allocation. YOLO11^27^ introduces the Cross-Stage Partial Spatial Attention (C2PSA) module to selectively focus on critical image regions, thereby improving accuracy in complex backgrounds. YOLO12^28^ shifts toward an attention-centric framework by proposing the Area Attention Module (A2) and R-ELAN, which maintain a large receptive field while minimizing computational complexity and memory footprint. Furthermore, YOLO13^29^ employs Hypergraph-Enhanced Adaptive Visual Perception (HyperACE) to model global high-order semantic relationships, dynamically optimizing performance for robust detection in complex scenarios.

### End-to-end object detectors

DETR^30^ was the first to introduce the Transformer encoder-decoder architecture, transforming object detection into an end-to-end set prediction task, eliminating traditional anchor box generation and Non-Maximum Suppression (NMS), and achieving fully automatic output through a one-to-one matching mechanism. Deformable DETR^31^ proposed a sparse attention mechanism, where attention is focused only on a small number of sampling locations around reference points, greatly improving small-target detection efficiency and significantly shortening training time. DINO^32^ integrates a hybrid query selection strategy and deformable attention, and demonstrates that noise injection and optimization during training can improve performance. RT-DETR^33^ targets real-time performance, introducing a hybrid encoder that decouples intra-scale information interaction and cross-scale fusion mechanisms, improving multi-scale feature processing efficiency and achieving an excellent balance between accuracy and speed. DEIM^34^ proposes an efficient Transformer-based object detection training method, with its core being Dense One-to-One (O2O) Matching. This method enhances the density of supervision signals by increasing the number of positive samples in each image, and uses Matchability-Aware Loss (MAL) to distinguish and optimize matching quality, thereby accelerating convergence while maintaining or further improving detection performance.

### Vision-based state space models

Based on research on State Space Models (SSMs), Mamba exhibits the advantage of linear complexity with respect to input size and solves the computational efficiency issue of Transformers in long-sequence state space modeling. In the field of general visual backbones, Vision Mamba^35^ proposes a pure visual backbone model based on selective SSMs, marking the first introduction of Mamba into the visual domain. VMamba^36^ introduces a Cross-Scan module, enabling the model to perform 2D image selective scanning, enhancing visual processing capabilities, and demonstrating superiority in image classification tasks. LocalMamba^37^ focuses on windowed scanning strategies for visual space models, optimizes visual information to capture local dependencies, and introduces a dynamic scanning method to search for optimal selections for different layers. Mamba YOLO^38^ proposes a new SSM model that does not require pre-training on large-scale datasets (e.g., ImageNet^39^). It designs LSBlock and RGBlock modules to enable the model to more accurately capture local dependencies in images, significantly enhancing model robustness and adapting specifically to object detection tasks, thereby demonstrating the potential of SSMs in object detection.

## Method

### Preliminaries

The structured state space sequence models, S4^40^ and Mamba^41^, originate from State Space Models (SSMs)^42^. Both are derived from a continuous system that maps a univariate sequence $x(t) \in \mathbb {R}$ to an output sequence $y(t)$ via an implicit intermediate state $h(t) \in \mathbb {R}^N$. This design not only establishes the relationship between the input and output but also encapsulates temporal dynamics. The system can be defined using the following mathematical equations:

1 $$\begin{aligned} h'(t) = A h(t) + B x(t) \end{aligned}$$

2 $$\begin{aligned} y(t) = C h(t) \end{aligned}$$

In Equation (1), $A \in \mathbb {R}^{N \times N}$ denotes the state transition matrix, which governs the evolution of the hidden state over time, while $B \in \mathbb {R}^{N \times 1}$ represents the weight matrix that associates the input space with the hidden state. Additionally, $C \in \mathbb {R}^{N \times 1}$ is the observation matrix, which maps the hidden intermediate state to the output. Mamba applies this continuous system to discrete-time sequence data by adopting fixed discretization rules to convert parameters $A$ and $B$ into their discrete counterparts $\tilde{A}$ and $\tilde{B}$, thereby integrating the system more effectively into deep learning architectures. A commonly used discretization method for this purpose is the Zero-Order Hold (ZOH). The discretized version can be defined as follows:

3 $$\begin{aligned} \tilde{A} = \exp (\Delta A) \end{aligned}$$

4 $$\begin{aligned} \tilde{B} = (\Delta A)^{-1} \left( \exp (\Delta A) - I \right) \Delta B \end{aligned}$$

In Equation (4), $\Delta$ represents a time-scale parameter that adjusts the temporal resolution of the model, and $\Delta A$ and $\Delta B$ denote the discrete-time counterparts of the continuous parameters within a given time interval. Here, $I$ denotes the identity matrix. After the transformation, the model performs computations in a linear recursive form, which can be defined as:

5 $$\begin{aligned} h'(t) = \tilde{A} h_{t-1} + \tilde{B} x_t \end{aligned}$$

6 $$\begin{aligned} y_t = C h_t \end{aligned}$$

The entire sequence transformation can also be expressed in a convolutional form, defined as follows:

7 $$\begin{aligned} \textbf{K} = \left( C \tilde{B}, C \tilde{A} \tilde{B}, \dots , C \tilde{A}^{L-1} \tilde{B} \right) \end{aligned}$$

8 $$\begin{aligned} y = x *\tilde{K} \end{aligned}$$

where $\tilde{K} \in \mathbb {R}^L$ denotes the structured convolution kernel, and $L$ represents the length of the input sequence. In the design proposed in this paper, the model adopts the convolutional form for parallel computation.

### The overall architecture of the model

Existing object detection models still face several limitations in feature extraction and computational efficiency. On the one hand, CNN-based architectures suffer from a limited receptive field, while Transformer-based architectures have insufficient capability for local feature extraction. As a result, the models struggle to capture small-object features effectively, leading to low feature utilization, and redundant features further increase model complexity. On the other hand, in the deep feature extraction stage, the attention mechanism of C2PSA is restricted, resulting in insufficient focus in complex scenes. Moreover, shallow features containing rich details are underutilized, which weakens small-object detection performance. In addition, the detection head contains a large number of parameters, resulting in excessive computational costs that hinder deployment on edge devices.

**Fig. 1:**
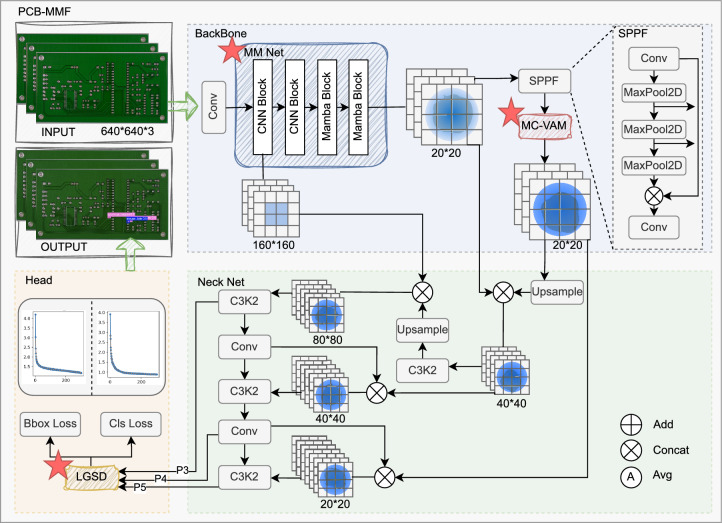
The overall architecture of the proposed PCB-MMF model.

To address these issues, this paper proposes a hybrid CNN–Mamba architecture for PCB defect detection, named PCB-MMF, which is built upon the single-stage detector YOLO11. The overall structure of PCB-MMF is illustrated in Fig. 1.

The proposed model consists of three key components: the backbone, the neck, and the detection head, which collaboratively enable efficient and accurate defect detection. Specifically, the backbone adopts a hybrid CNN–Mamba structure to extract multi-level features. By integrating both local and global information, the backbone enhances the representation capability for small objects and complex backgrounds, thereby improving robustness. The neck further aggregates the multi-scale features from the backbone and passes them through the Spatial Pyramid Pooling Fast (SPPF) module into the Multi-Cognition Visual Attention Module (MC-VAM). By leveraging multi-cognition feature fusion and deep residual connections, MC-VAM increases the utilization of shallow features, allowing the model to focus more effectively on core defect regions and improve multi-scale perception. Finally, the detection head employs a lightweight structure named LGSD, which is based on Group Normalization and shared convolution. This design ensures effective feature fusion while significantly reducing parameter size and computational complexity, making the model more suitable for deployment on edge devices.

### MM NET

**Fig. 2:**
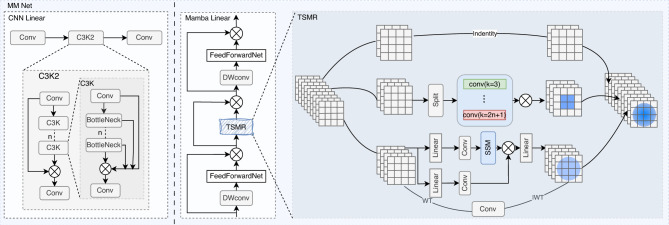
The architecture of the proposed MM Net.

To enhance the ability of the model to capture small-object features, we design a hybrid feature extraction network named MM Net, which integrates CNN and Mamba architectures. The structure of MM Net is illustrated in Fig. 2.

MM Net consists of two main components: the CNN layer and the Mamba layer. The CNN layer focuses on extracting local features, while the Mamba layer is responsible for modeling global feature dependencies.

The Mamba layer is a lightweight structure that combines multi-scale convolutional feature extraction with sequence modeling, aiming to capture both local spatial patterns and long-range dependencies.

Specifically, the Mamba layer introduces a Three-Stage Multi-receptive-field fusion module (TSMR), which partitions the input channels (denoted as $c$) into three branches: a global branch (ratio $\xi$, $0< \xi < 1$), a local branch (ratio $\mu$, $0< \mu < \xi$), and an identity mapping branch (ratio $1 - \xi - \mu$)^43^.

It is important to emphasize that TSMR is designed as an integral, built-in structural component of the Mamba layer rather than an optional add-on block. Therefore, whenever the MM-NET backbone is utilized, the TSMR mechanism is inherently active.

In the global branch, MBWTConv2d^44^ based on wavelet transform is combined with a state-space model (SSM) containing symmetric sub-branches to enhance long-range dependency modeling. The local branch adopts depthwise separable convolutions (DWConv2d) to preserve fine-grained local textures, where *j* denotes the *j*-th convolution layer and $n$ (set to 3 in our experiments) is the total number of layers.

The output of the local branch is computed as follows:

9 $$\begin{aligned} \textbf{x}^O_{\text {L}j} = \operatorname {Conv}(\textbf{x}^I_{\text {L}j}, k = 2j + 1), \quad j \in \{1, \dots , n\} \end{aligned}$$

10 $$\begin{aligned} \textbf{x}^O_{\text {L}} = \operatorname {Concat}([\textbf{x}^O_{\text {L}1}, \dots , \textbf{x}^O_{\text {L}n}], \text {dim}=-1) \end{aligned}$$

For the remaining $(1 - \xi - \mu )c$ channels, an identity mapping branch is applied to reduce redundancy. The outputs of the three branches are then fused along the channel dimension and re-projected via a convolutional layer.

To further strengthen the feature representation, residual depthwise convolutions and a Feed-Forward Network (FFN) are embedded before and after the Mamba layer, along with residual connections and a DropPath mechanism, which enhance training stability and generalization.

This design achieves efficient multi-scale feature fusion and global–local collaborative modeling while maintaining low computational complexity, making it suitable for real-time visual tasks.

The final output of the MM Net is expressed as:

11 $$\begin{aligned} \textbf{x}^O = \operatorname {Concat}(\textbf{x}^O_{\text {G}}, \textbf{x}^O_{\text {L}}, \textbf{x}^I[(1 - \xi - \mu )c:]) \end{aligned}$$

### MC-VAM

**Fig. 3:**
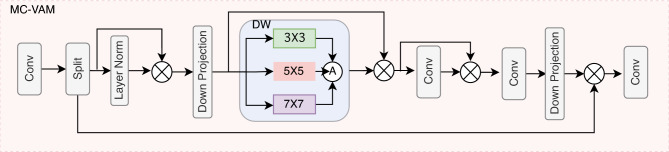
The architecture of the proposed MC-VAM module.

To address the insufficient attention to complex scenes and the underutilization of shallow features in the deep feature extraction stage, which often lead to high information loss, we propose the Multi-Cognition Visual Attention Module (MC-VAM). Its architecture is illustrated in Fig. 3.

MC-VAM enhances the model’s capability of handling complex scenarios through a multi-scale feature extraction design. Specifically, the input features are first normalized using Layer Normalization (LN) and adjusted by two learnable scaling factors, $s_{1}$ and $s_{2}$^45^. The normalization process is formulated as:

12 $$\begin{aligned} X_{norm} = s_{1} \odot \text {LN}(X_{in}) + s_{2} \odot X_{in} \end{aligned}$$

The normalized features are then projected into a lower-dimensional space via a $1 \times 1$ convolution to reduce computational overhead:

13 $$\begin{aligned} X_{down} = W_{down} *X_{norm} \end{aligned}$$

After dimensionality reduction, the features are processed by three parallel depthwise separable convolutions (DWConv) with varying kernel sizes ($3 \times 3$, $5 \times 5$, and $7 \times 7$) to capture multi-scale receptive fields. A residual connection is added to preserve original information. The outputs of these parallel branches are aggregated by averaging:

14 $$\begin{aligned} X_{dw} = X_{down} + \frac{1}{3} \sum _{k \in \{3,5,7\}} ( W_{dw}^{k \times k} *X_{down} ) \end{aligned}$$

To facilitate cross-channel information interaction, the aggregated features pass through a pointwise convolution ($1 \times 1$):

15 $$\begin{aligned} X_{pw} = X_{dw} + W_{pw} *X_{dw} \end{aligned}$$

Finally, the features are activated by the Gaussian Error Linear Unit (GELU), upsampled to restore the original spatial dimensions, and fused with the initial input via a deep residual connection:

16 $$\begin{aligned} X_{out} = X_{in} + \operatorname {Up}( \operatorname {GELU}(X_{pw}) ) \end{aligned}$$

To ensure clarity and reproducibility, all mathematical symbols utilized in the MC-VAM equations are defined in Table 1.

In addition, MC-VAM incorporates deep residual connections throughout the module to strengthen the utilization of shallow features and mitigate feature loss. This design improves the perception of complex scenes and small objects while maintaining low parameter overhead.

**Table 1:** Nomenclature of symbols used in the MC-VAM mathematical formulation.

Symbol	Definition
$X_{in}, X_{out}$	The input and output feature maps of the MC-VAM module.
$X_{norm}, X_{down}, X_{dw}, X_{pw}$	Intermediate feature maps generated at corresponding stages.
$\text {LN}(\cdot )$	Layer Normalization operation.
$s_{1}, s_{2}$	Learnable channel-wise scaling factors.
$\odot$	Element-wise multiplication (Hadamard product).
$*$	Convolution operation.
$W_{down}, W_{pw}$	Weights of the $1 \times 1$ down-projection and pointwise convolutions, respectively.
$W_{dw}^{k \times k}$	Weights of the depthwise convolution with kernel size $k \times k$.
$\operatorname {GELU}(\cdot )$	GELU non-linear activation function.
$\operatorname {Up}(\cdot )$	Upsampling operation to restore spatial dimensions.
$\xi , \mu$	Channel ratio of global/local branch in TSMR module ($0< \mu< \xi < 1$).
$c$	Number of input feature map channels (positive integer).
$n$	Number of convolution layers in local branch (set to 3 in experiments).

### LGSD

**Fig. 4:**
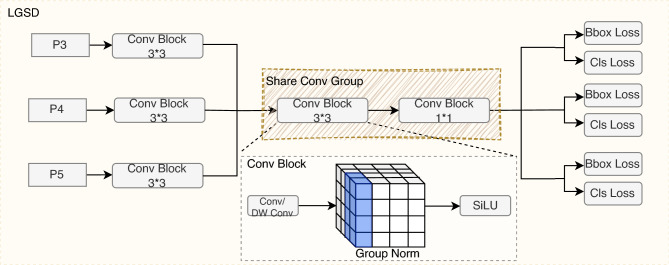
Illustration of the LGSD detection head.

Balancing detection accuracy and computational efficiency in multi-scale object detection remains a major challenge. To address this, we propose a lightweight detection head named LGSD, which integrates Group Normalization (GN)^46^ and shared convolutions. As illustrated in Fig. 4, LGSD significantly reduces the number of parameters and computational cost while maintaining high detection accuracy, making it well-suited for deployment on resource-constrained devices (Fig. 4).

Specifically, the LGSD head operates on the multi-scale feature maps output by the neck network. For each feature map, an independent $3 \times 3$ convolution block is first applied to align the channel dimensions and semantic distribution across scales. Then, all detection branches share a convolution group based on depthwise separable convolutions, where depthwise convolution captures local spatial patterns within each channel and pointwise convolution ($1 \times 1$) enables inter-channel feature interaction. To stabilize feature distribution, Group Normalization is employed, defined as follows:

17 $$\begin{aligned} \mu _i = \frac{1}{m} \sum _{k \in S_i} x_k, \end{aligned}$$

18 $$\begin{aligned} \sigma _i = \sqrt{\frac{1}{m} \sum _{k \in S_i} (x_k - \mu _i)^2 + \epsilon }, \end{aligned}$$

where $x_k$ denotes an element of the input feature $x \in \mathbb {R}^{C \times H \times W}$ belonging to the *i*-th group $S_i$, *m* is the group size, $\mu _i$ and $\sigma _i$ are the mean and standard deviation, $\epsilon$ is a small constant to prevent division by zero, and $\gamma$ and $\beta$ are learnable affine parameters.

After normalization and non-linear activation, the features are divided into two detection branches: the regression branch and the classification branch. The regression branch employs a $1 \times 1$ convolution to predict bounding box parameters, with a learnable scaling factor for adaptive adjustment. The classification branch predicts class probabilities using an independent $1 \times 1$ convolution. The outputs of the two branches are concatenated along the channel dimension to form the final detection prediction. This design not only reduces redundant computation but also enhances semantic consistency across scales through shared convolutions, achieving a balance between lightweight efficiency and detection accuracy.

## Experiment

### Datasets

To evaluate the effectiveness of the proposed method, three publicly available PCB defect detection datasets were selected. We conducted comprehensive comparisons with state-of-the-art models under the same experimental environment, and performed ablation studies on all three datasets to validate the effectiveness and generalizability of our proposed modules.

#### HRIPCB dataset

**Fig. 5:**
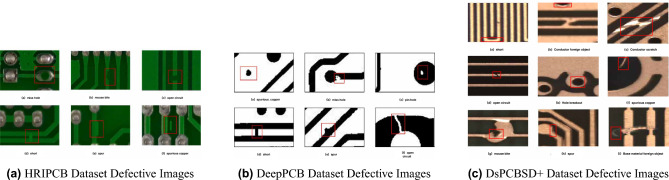
Defective Images from HRIPCB, DeepPCB, and DsPCBSD+ Datasets.

The HRIPCB dataset, proposed by Peking University’s Intelligent Robotics Laboratory^47^, contains 693 defect images with six defect types: missing hole, mouse bite, open, short, spur, and copper (as shown in Fig. 5a). Each image contains approximately 3–5 defects, and the defect categories are relatively balanced, which mitigates class imbalance issues. We randomly split the dataset into training, validation, and test sets in a 7:2:1 ratio.

#### DeepPCB dataset

The DeepPCB dataset, published by the Institute of Image Processing and Pattern Recognition, Shanghai Jiao Tong University^48^, contains 1500 pairs of images. Each pair consists of a defect image and a corresponding defect-free reference image. Six common defects are annotated, including open, short, mouse bite, spur, copper, and pin-hole (as shown in Fig. 5b). To address the scarcity of real defects, some synthetic defects were included. All images are binarized to reduce the influence of illumination and background noise. The dataset was split into training, validation, and test sets with a 7:2:1 ratio.

#### DsPCBSD + dataset

The DsPCBSD+ dataset, collected and released by South China Agricultural University^49^, contains 10,259 real PCB defect images with 20,276 manually annotated bounding boxes. Defects are categorized into four main groups (copper defects, missing material, scratches, and foreign objects) and further divided into nine specific subtypes: short, spur, spurious copper, open, mouse bite, hole breakout, conductor scratch, conductor foreign object and base material foreign object (as shown in Fig. 5c). Unlike the previous datasets, DsPCBSD+ incorporates more diverse and challenging defect types such as hole breakout, scratches, and foreign objects, which are often overlooked in other datasets but play a critical role in practical industrial quality inspection. The dataset was split into training, validation, and test sets in a 7:2:1 ratio.

**Table 2:** Detailed statistical information of the three experimental datasets.

Dataset	Defect category	Label	Instances	Total images	Total instances
HRIPCB	Missing hole	Missing hole	497	693	2953
	Mouse bite	Mouse bite	492		
	Open circuit	Open circuit	482		
	Short	Short	491		
	Spur	Spur	488		
	Spurious copper	Spurious copper	503		
DeepPCB	Open circuit	open	1702	1500	8853
	Short circuit	short	1317		
	Mouse bite	mousebite	1748		
	Spur	spur	1445		
	Spurious copper	copper	1,321		
	Pin-hole	pin-hole	1320		
DsPCBSD+	Short	SH	915	10,259	20,276
	Spur	SP	4584		
	Spurious copper	SC	1593		
	Open circuit	OP	1770		
	Mouse bite	MB	2529		
	Hole breakout	HB	2883		
	Conductor scratch	CS	2490		
	Conductor foreign object	CFO	1832		
	Base material foreign object	BMFO	1680		

The detailed category distribution for each dataset is summarized in Table 2. In this study, we adopted a 7:2:1 ratio for training, validation, and testing. While an 8:1:1 split is common in large-scale datasets, the 7:2:1 allocation is equally robust and widely favored in specialized PCB research. Specifically for smaller datasets like HRIPCB (693 images), a 20% validation set provides a more representative and statistically stable sample for hyperparameter tuning and early stopping, thereby ensuring the structural stability and generalization of the PCB-MMF model.

#### Data augmentation

**Fig. 6:**
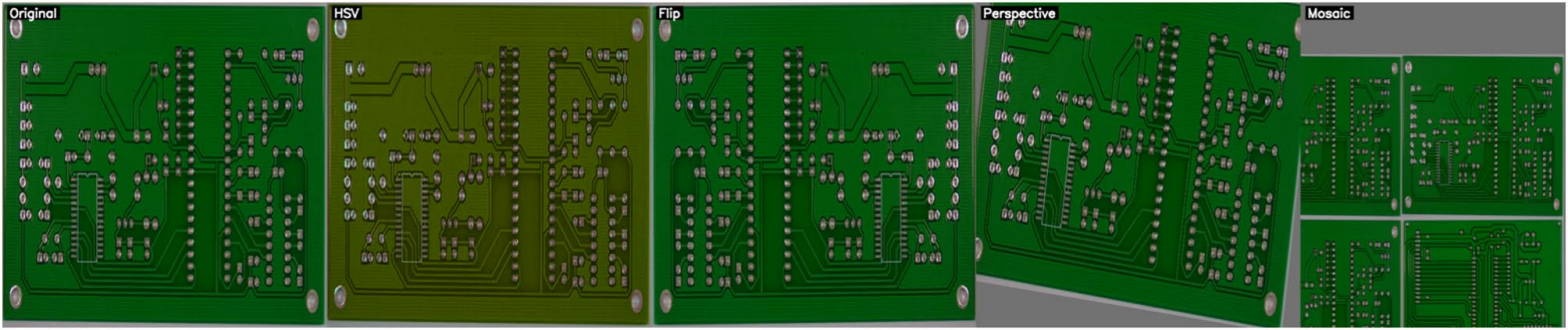
Pictorial representation of the data augmentation methods used in this study, including original image, HSV adjustment, spatial flipping, perspective transformation, and Mosaic augmentation.

To enhance the robustness of the model and improve its ability to detect defects under varying industrial conditions, a diverse data augmentation pipeline was applied during the training phase. As shown in Fig. 6, the primary methods include HSV color space adjustment, spatial flipping, perspective transformation, and Mosaic augmentation.

The HSV adjustment simulates various lighting conditions in PCB manufacturing, while spatial flipping and perspective transformations account for different board orientations and camera angles. Notably, Mosaic augmentation combines four training images into one, effectively increasing the complexity of the background and significantly enhancing the model’s sensitivity to small-scale defects. This comprehensive augmentation strategy ensures that the proposed PCB-MMF framework maintains stable and high-precision performance across all three datasets.

### Experimental environment and settings

The experimental platform for this study was built on an Ubuntu 22.04 operating system, equipped with an Intel Xeon(R) Platinum 8352V CPU and an NVIDIA RTX 3090 GPU. The implementation was conducted using the PyTorch 2.1.0 framework with CUDA 12.1 for hardware acceleration.

To ensure complete transparency and reproducibility, all experimental configurations—including hardware specifications, software environments, and training hyperparameters—are explicitly consolidated in Table 3. Several key optimization strategies were designed to enhance the model’s stability and accuracy. Specifically, we employed the Stochastic Gradient Descent (SGD) optimizer with an initial learning rate of 0.01 and a momentum of 0.937. The training process spanned 300 epochs, incorporating a 3-epoch linear warmup strategy to stabilize initial gradients and prevent early-stage divergence. The total loss function is formulated as a weighted fusion of Bounding Box Regression Loss (CIoU), Classification Loss (BCE), and Distribution Focal Loss (DFL), strictly penalizing localization errors to ensure highly precise bounding boxes for microscopic PCB defects.

Furthermore, to effectively mitigate the potential risks of overfitting and class imbalance inherent in datasets with relatively small sample sizes (e.g., the HRIPCB dataset), Mosaic data augmentation was heavily utilized. By randomly scaling, cropping, and stitching four distinct images into a single synthetic input, Mosaic augmentation exponentially increases batch diversity and global contextual awareness. Crucially, we implemented a “Close-Mosaic” strategy during the final 10 epochs. Disabling heavy augmentation in the late training stage allows the model to transition from learning complex synthetic scenes to fine-tuning strictly on the true, unaugmented data distribution, thereby significantly boosting the final detection robustness and generalization capability.

**Table 3:** Consolidated experimental environment, training hyperparameters, and evaluation configurations.

Category	Parameter	Value / specification
Hardware Environment	Operating System	Ubuntu 22.04
	CPU	Intel Xeon(R) Platinum 8352V
	GPU	NVIDIA RTX 3090
Software Framework	Deep Learning Library	PyTorch 2.1.0
	Acceleration Toolkit	CUDA 12.1
Training Basics	Input Image Size (*imgsz*)	$640 \times 640$
	Batch Size (*batch*)	32
	Total Epochs (*epochs*)	300
	Optimizer	SGD
Learning Rate & Optimization	Initial Learning Rate ($lr_0$)	0.01
	Final Learning Rate Fraction (*lrf*)	0.01
	Momentum (*momentum*)	0.937
	Weight Decay ($weight\_decay$)	0.0005
Warmup Strategy	Warmup Epochs	3.0
	Warmup Momentum	0.8
	Warmup Initial Bias LR	0.1
Data Augmentation	Mosaic Probability	1.0
	Close Mosaic (last *N* epochs)	10
Evaluation & NMS	Confidence Threshold	0.001
	IoU Threshold (NMS)	0.7
	Multi-Scale Testing	Disabled

### Evaluation metrics

The experimental evaluation metrics include Precision (*P*), Recall (*R*), Average Precision (*AP*), and mean Average Precision (*mAP*). To provide a clear focal point, we explicitly define **mAP@50** as the primary evaluation metric for detection accuracy, while treating the others as supplementary indicators. Among these, the calculation formulas for Precision, Recall, AP, and mAP are as follows:The experimental evaluation metrics include Precision (*P*), Recall (*R*), Average Precision (*AP*), mean Average Precision (*mAP*), number of parameters (Params), and computational complexity (GFLOPs). Additionally, to comprehensively assess deployment efficiency, Model Size (MB) and inference speed (FPS) are also evaluated. Among these, the calculation formulas for Precision, Recall, AP, and mAP are as follows:

19 $$\begin{aligned} P = \frac{TP}{TP + FP} \end{aligned}$$

20 $$\begin{aligned} R = \frac{TP}{TP + FN} \end{aligned}$$

21 $$\begin{aligned} AP = \int _{0}^{1} P(R) dR \end{aligned}$$

22 $$\begin{aligned} mAP = \frac{1}{N} \sum _{i=1}^{n} AP_i \end{aligned}$$

### Comparative experiment

In the category of one-stage detectors, the YOLO family (e.g., v5/v8) leverages the CSPDarknet backbone, PANet for feature fusion, and CIoU loss to achieve efficient detection, while YOLO11/12/13 further introduce Transformer modules and spatial-channel decoupled downsampling to enhance global modeling capability and computational efficiency. Transformer-based detectors such as RT-DETR adopt global attention mechanisms to achieve end-to-end detection without NMS post-processing, demonstrating strong robustness in PCB images with complex backgrounds but incurring high training costs. Moreover, hybrid architectures such as Mamba-YOLO replace part of the convolutional layers with state-space models (SSMs), significantly improving long-sequence feature modeling but at the expense of higher computational complexity.

**Fig. 7:**
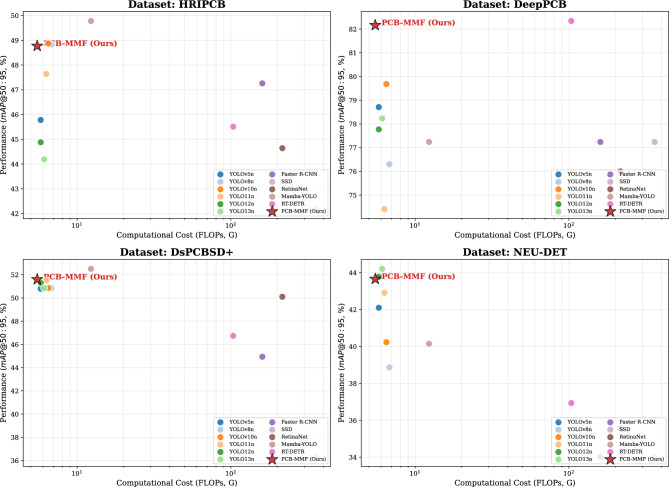
Optimal Balance Analysis: Accuracy vs. Efficiency across Four Datasets.

To thoroughly validate the superiority of the proposed PCB-MMF model and ensure a rigorous “apples-to-apples” comparison, we conducted comparative experiments with the aforementioned mainstream detectors under strictly identical experimental settings. Crucially, all baseline models—including MMDetection-based classical detectors (Faster R-CNN, SSD, and RetinaNet)—were re-trained or reproduced using their official codebases under the unified hyperparameter recipe detailed in Table 3. This methodology guarantees that the observed performance gains are strictly attributable to our architectural innovations rather than inconsistent training setups. Figure 7 intuitively demonstrate different aspects of the comparative experiments.

On the HRIPCB dataset, YOLO series models exhibited a trade-off between precision and recall: YOLOv8 achieved the highest recall (90.75%), while YOLOv10 obtained slightly better mAP@50:95 (48.87%). Mamba-YOLO demonstrated advantages in mAP@50 (92.87%) and mAP@75 (47.45%) due to sequence modeling but required substantially higher computational costs. In contrast, the proposed PCB-MMF achieved the highest precision (95.97%) and mAP@50 (93.43%) with only 5.5G FLOPs and approximately 2.35M parameters. This performance gain can be attributed to the MM-NET network and the TSMR module, which jointly enhance the ability to capture fine-grained PCB defect features while maintaining low complexity (see Table 4).

**Table 4:** Comparative experiments on the HRIPCB dataset.

Model	Precision (%)	Recall (%)	mAP50 (%)	mAP75 (%)	mAP50-95 (%)	Flops (G)	Params (M)	FPS	Size (M)
YOLOv5n	93.74	83.78	90.22	37.28	45.78	*5.8*	**2.18**	*74.85*	**4.5**
YOLOv8n	93.88	90.75	92.21	44.22	48.83	6.8	2.69	**75.04**	5.4
YOLOv10n	93.12	84.24	91.17	41.07	*48.87*	6.5	*2.27*	66.15	5.5
YOLO11n	92.18	86.71	90.55	40.16	47.64	6.3	2.58	65.32	5.3
YOLO12n	92.99	83.07	87.73	39.03	44.88	*5.8*	2.51	46.23	5.2
YOLO13n	93.47	84.66	89.13	35.59	44.19	6.1	2.45	36.53	5.2
Faster R-CNN	94.04	**93.44**	91.90	*45.19*	47.26	160.15	28.15	32.42	215.5
SSD	90.01	88.78	86.63	37.91	42.18	360.80	24.41	40.98	186.3
RetinaNet	*95.40*	*91.04*	91.14	37.92	44.64	216.1	36.21	10.32	277.5
Mamba-YOLO-T	93.41	87.45	*92.87*	**47.45**	**49.78**	12.3	5.66	30.44	11.2
RT-DETR-L	85.12	86.49	91.4	36.13	45.51	103.5	32.00	23.33	63.1
PCB-MMF	**95.97**	86.78	**93.43**	44.71	48.77	**5.5**	2.35	50.64	*4.8*

Bold indicates the best performance, and italics indicates the second-best performance.

On the DeepPCB dataset, YOLOv10 achieved strong results in precision (97.00%) and mAP@50:95 (79.68%), while YOLOv5 balanced precision (96.88%) and recall (95.70%). Mamba-YOLO also performed competitively in recall and mAP@50, albeit with higher computational overhead. RT-DETR achieved the best performance across most metrics (Precision = 98.20%, Recall = 97.66%, mAP@50 = 98.74%, mAP@75 = 94.65%), but its complexity (103.5G FLOPs,  32M parameters) makes deployment impractical. PCB-MMF, enhanced with the MC-VAM module for multi-scale feature fusion and residual connections to preserve shallow features, achieved competitive results (mAP@50:95=82.16%) with only 5.5G FLOPs and 2.36M parameters, demonstrating an optimal balance between efficiency and accuracy (see Table 5).

**Table 5:** Comparative experiments on the DeepPCB dataset.

Model	Precision (%)	Recall (%)	mAP50 (%)	mAP75 (%)	mAP50-95 (%)	Flops (G)	Params (M)	FPS	Size (M)
YOLOv5n	96.88	95.70	98.35	92.39	78.71	*5.8*	**2.18**	*103.24*	**4.5**
YOLOv8n	95.60	96.21	98.49	88.51	76.30	6.8	2.69	**108.26**	5.4
YOLOv10n	*97.00*	94.85	98.46	92.70	79.68	6.5	*2.27*	94.04	5.5
YOLO11n	96.36	95.95	98.17	88.54	74.41	6.3	2.58	89.98	5.3
YOLO12n	*97.00*	94.68	98.10	92.14	77.77	*5.8*	2.51	52.95	5.2
YOLO13n	96.40	94.29	97.96	91.77	78.23	6.1	2.45	40.53	5.2
Faster R-CNN	96.80	95.96	98.37	89.54	77.24	160.15	28.15	26.42	215.5
SSD	96.80	95.96	98.37	89.54	77.24	12.3	5.66	34.75	186.3
RetinaNet	96.10	90.01	98.30	91.51	76.00	216.1	36.21	12.13	277.5
Mamba-YOLO-T	96.80	95.96	98.37	89.54	77.24	12.3	5.66	36.32	11.2
RT-DETR-L	**98.20**	**97.66**	**98.74**	**94.65**	**82.34**	103.5	32.00	30.00	63.1
PCB-MMF	96.23	*97.02*	*98.68*	*94.56*	*82.16*	**5.5**	2.36	73.79	*4.8*

Bold indicates the best performance, and italics indicates the second-best performance.

On the DsPCBSD+ dataset, which contains highly diverse defects, YOLO series models showed performance degradation, with YOLOv11 performing best (mAP@50:95 = 51.51%). Mamba-YOLO achieved higher mAP@75 (54.83%) and mAP@50:95 (52.51%), highlighting its adaptability in complex scenarios, whereas RT-DETR showed significant performance degradation (mAP@50:95 = 46.74%), indicating insufficient generalization to texture-rich defects. PCB-MMF, benefiting from the LGSD detection head, achieved competitive performance in precision (81.85%), recall (79.69%), mAP@50 (85.39%), and mAP@50:95 (51.60%), while maintaining the lowest computational cost, further validating its robustness and practicality for industrial PCB defect detection (see Table 6).

**Table 6:** Comparative experiments on the DsPCBSD+ dataset.

Model	Precision (%)	Recall (%)	mAP50 (%)	mAP75 (%)	mAP50-95 (%)	Flops (G)	Params (M)	FPS	Size (M)
YOLOv5n	*82.93*	78.92	84.38	53.13	50.79	*5.8*	**2.18**	*104.11*	**4.5**
YOLOv8n	81.25	79.58	83.91	52.28	50.85	6.8	2.69	**106.56**	5.4
YOLOv10n	82.68	77.42	84.61	52.86	50.86	6.5	*2.27*	98.12	5.5
YOLO11n	**83.64**	77.49	84.78	53.54	51.51	6.3	2.58	96.64	5.3
YOLO12n	81.70	79.47	84.16	52.85	51.30	*5.8*	2.51	58.84	5.2
YOLO13n	81.59	78.38	83.59	53.19	50.85	6.1	2.45	44.27	5.2
Faster R-CNN	66.82	**87.50**	82.10	42.59	44.94	160.15	28.15	28.69	215.6
SSD	75.57	71.56	73.84	29.39	36.31	362.52	24.82	30.11	186.3
RetinaNet	81.88	80.49	83.21	50.92	50.10	216.1	36.21	11.26	277.5
Mamba-YOLO-T	82.45	*80.94*	**85.58**	**54.83**	**52.51**	12.3	5.66	36.46	11.2
RT-DETR-L	77.72	73.63	79.29	49.00	46.74	103.5	32.00	28.34	63.1
PCB-MMF	81.85	79.69	*85.39*	*53.65*	*51.60*	**5.5**	2.36	78.42	*4.8*

Bold indicates the best performance, and italics indicates the second-best performance.

### Ablation experiment

To systematically evaluate the individual contribution of each proposed component, we conducted comprehensive ablation studies across the HRIPCB, DeepPCB, and DsPCBSD+ datasets. The baseline model is YOLO11, to which we progressively integrated the MM-NET backbone, the MC-VAM module, and the LGSD detection head. Note that in these experiments, the TSMR module is evaluated as an inseparable, core component of the MM-NET backbone. Thus, the performance gains attributed to MM-NET inherently reflect the contribution of the TSMR-embedded Mamba layers across all experimental scenarios.

On the HRIPCB dataset, the results demonstrate that all three proposed modules consistently contribute to performance improvements. With MM-NET, recall increased to 88.53% and mAP@75 to 43.97%, indicating that the module effectively combines global and fine-grained local features to enhance small-defect detection. Incorporating MC-VAM further improved mAP@50 to 91.56% by adaptively weighting multi-scale features, while residual connections preserved shallow features. Adding the LGSD detection head raised precision to 94.15% and mAP@0.5:0.95 to 47.83%, while reducing parameters from 2.58 to 2.42M. It is worth noting that while the configuration without LGSD achieves a slightly higher mAP@50:95 (49.32%), the full model was selected as the final architecture to prioritize practical deployability on edge devices. By employing shared convolutions, the LGSD module intentionally trades a minor drop in this comprehensive metric for substantial efficiency gains—reducing the overall parameter count to 2.35M and FLOPs to 5.5G. Consequently, the full PCB-MMF model delivers the optimal trade-off between inspection accuracy and computational cost (see Table 7).

**Table 7:** Ablation experiments on the HRIPCB dataset.

YOLO11N	MM NET	MC-VAM	LGSD	Precision (%)	Recall (%)	mAP50 (%)	mAP75 (%)	mAP50-95 (%)	Flops (G)	Params (M)
$\checkmark$				92.18	86.71	90.55	40.16	47.64	6.3	2.58
$\checkmark$	$\checkmark$			89.13	*88.53*	91.83	*43.97*	48.24	6.2	2.46
$\checkmark$		$\checkmark$		92.78	85.41	91.56	41.87	48.64	6.4	2.64
$\checkmark$			$\checkmark$	94.15	87.62	92.58	36.81	47.83	*5.6*	*2.42*
$\checkmark$	$\checkmark$	$\checkmark$		*95.17*	**88.81**	**93.77**	41.21	**49.32**	6.2	2.51
$\checkmark$	$\checkmark$	$\checkmark$	$\checkmark$	**95.97**	86.78	*93.43*	**44.71**	*48.77*	**5.5**	**2.35**

Bold indicates the best performance, and italics indicates the second-best performance

On the DeepPCB dataset, although the baseline already performed well, the modules further improved results. MM-NET raised recall to 94.69% and mAP@75 to 92.70%, while MC-VAM boosted mAP@50 to 98.61%, enhancing performance in complex regions. The LGSD detection head reduced parameters without compromising accuracy. When integrated, the three modules achieved recall of 97.02% and mAP@75 of 94.56%, showcasing their complementary nature (see Table 8).

**Table 8:** Ablation experiments on the DeepPCB dataset.

YOLO11N	MM NET	MC-VAM	LGSD	Precision (%)	Recall (%)	mAP50 (%)	mAP75 (%)	mAP50-95 (%)	Flops (G)	Params (M)
$\checkmark$				96.36	95.95	98.17	88.54	74.41	6.3	2.58
$\checkmark$	$\checkmark$			97.13	94.69	98.54	92.70	78.98	6.2	2.46
$\checkmark$		$\checkmark$		96.84	95.79	98.61	92.12	77.73	6.4	2.64
$\checkmark$			$\checkmark$	**98.51**	96.75	**98.87**	94.03	80.93	*5.6*	*2.42*
$\checkmark$	$\checkmark$	$\checkmark$		*97.32*	*96.98*	*98.82*	*94.49*	**82.55**	6.2	2.51
$\checkmark$	$\checkmark$	$\checkmark$	$\checkmark$	96.23	**97.02**	98.68	**94.56**	*82.16*	**5.5**	**2.36**

Bold indicates the best performance, and italics indicates the second-best performance.

On the DsPCBSD+ dataset, where the baseline performed relatively poorly (Precision 83.64%, mAP@50:95=51.51%), the modules provided notable improvements. MM-NET improved recall to 79.37% and mAP@50:95 to 51.92%; MC-VAM further raised recall to 79.91% and mAP@50:95 to 52.36%; LGSD improved recall to 80.65% and mAP@50:95 to 52.12% while reducing parameters. When combined, the three modules achieved mAP@0.5:0.95 of 51.60%, demonstrating balanced improvements despite dataset complexity (see Table 6).(see Table 9).

**Table 9:** Ablation experiments on the DsPCBSD+ dataset.

YOLO11N	MM NET	MC-VAM	LGSD	Precision (%)	Recall (%)	mAP50 (%)	mAP50-95 (%)	Flops (G)	Params (M)
$\checkmark$				**83.64**	77.49	84.78	51.51	6.3	2.58
$\checkmark$	$\checkmark$			*82.95*	79.37	85.18	51.92	6.2	2.46
$\checkmark$		$\checkmark$		81.63	*79.91*	**85.62**	**52.36**	6.4	2.64
$\checkmark$			$\checkmark$	81.82	**80.65**	*85.58*	*52.12*	*5.6*	*2.42*
$\checkmark$	$\checkmark$	$\checkmark$		82.63	78.40	84.64	51.62	6.2	2.51
$\checkmark$	$\checkmark$	$\checkmark$	$\checkmark$	81.85	79.69	85.39	51.60	**5.5**	**2.36**

Bold indicates the best performance, and italics indicates the second-best performance.

### Generalization evaluation

**Fig. 8:**
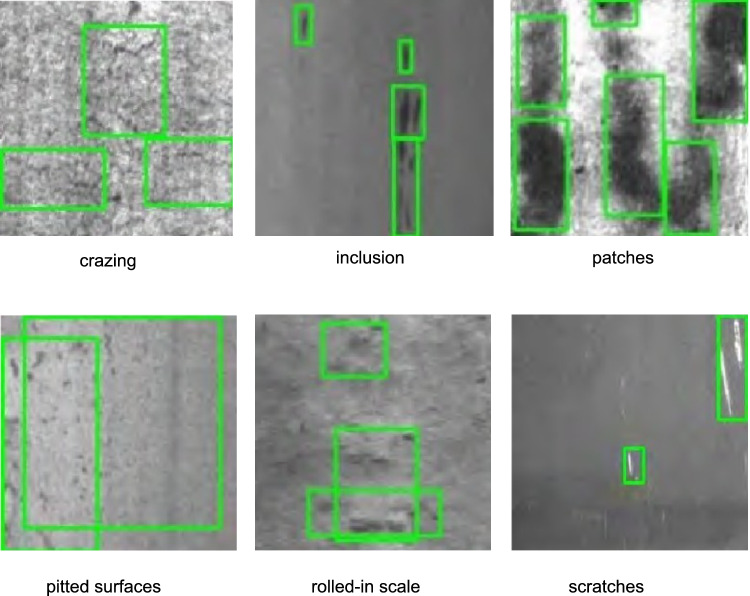
NEU-DET Dataset Defective Images.

To rigorously verify that the structural advantages of the proposed PCB-MMF architecture are not over-fitted to PCB-specific features, we extended our evaluation to a completely different industrial domain: the NEU-DET (Northeastern University Surface Defect Database) dataset. Unlike PCB imagery, NEU-DET comprises hot-rolled steel strip surface defects, presenting entirely distinct background textures, illumination variations, and defect morphologies (e.g., crazing, inclusion, patches, pitted surfaces, rolled-in scale, and scratches).Partial qualitative detection results are shown in Fig. 8.

We trained and evaluated the PCB-MMF model on the NEU-DET dataset using the exact same unified training recipe and hyperparameter configurations detailed in Table 3. The comprehensive quantitative results, comparing PCB-MMF against other state-of-the-art detectors on the NEU-DET dataset, are explicitly detailed in Table 10.

As the experimental data indicates, PCB-MMF achieves the highest detection accuracy with an mAP@50 of 76.69%, outperforming all evaluated models, including the latest YOLO variants (e.g., YOLO13 at 76.39%) and the SSM-based Mamba-YOLO (76.48%). Furthermore, it maintains highly competitive performance under stringent evaluation metrics, yielding an mAP@50:95 of 43.66% and an mAP@75 of 42.86%.

Beyond detection precision, PCB-MMF demonstrates exceptional lightweight characteristics and efficiency. It requires the lowest computational overhead among all compared models, utilizing only 5.5G FLOPs. Additionally, its parameter count (2.35M) and overall model size (4.8M) remain remarkably compact, ranking second only to the highly simplified YOLOv5 while delivering significantly superior accuracy. In terms of inference speed, PCB-MMF achieves 69.79 FPS, nearly doubling the processing speed of the Mamba-YOLO framework (37.20 FPS) and vastly outperforming traditional heavy-weight detectors such as Faster R-CNN and SSD.

This rigorous validation confirms that PCB-MMF proves to be not merely a PCB-specific detector, but a highly adaptable, highly efficient, and robust architectural solution for broader industrial surface defect detection tasks.

**Table 10:** Comparative experiments on the NEU-DET dataset.

Model	Precision (%)	Recall (%)	mAP50 (%)	mAP75 (%)	mAP50-95 (%)	Flops (G)	Params (M)	FPS	Size (M)
YOLOv5n	71.39	69.73	75.32	41.56	42.10	*5.8*	**2.18**	**124.75**	**4.5**
YOLOv8n	74.47	66.45	73.09	33.47	38.87	6.8	2.69	*112.12*	5.4
YOLOv10n	65.80	69.32	73.44	40.66	40.23	6.5	*2.27*	102.27	5.5
YOLO11n	74.12	70.51	75.10	42.80	42.91	6.3	2.58	102.43	5.3
YOLO12n	73.70	69.25	76.32	*43.74*	*43.78*	*5.8*	2.51	56.10	5.2
YOLO13n	73.89	68.59	76.39	**44.13**	**44.21**	6.1	2.45	42.77	5.2
Mamba-YOLO	**76.33**	69.79	*76.48*	34.12	40.15	12.3	5.66	37.20	11.2
RT-DETR	64.85	60.47	65.70	37.87	36.93	103.5	32.00	20.93	63.1
Faster R-CNN	48.01	**86.93**	71.92	30.05	34.01	160.15	28.15	22.15	215.50
SSD	70.47	71.23	71.82	27.85	34.02	360.80	24.41	34.55	186.30
Retinanet	67.52	*77.58*	71.17	31.50	35.57	216.1	36.21	10.03	277.5
PCB-MMF	*74.93*	68.69	**76.69**	42.86	43.66	**5.5**	2.35	69.79	*4.8*

Bold indicates the best performance, and italics indicates the second-best performance.

**Fig. 9:**
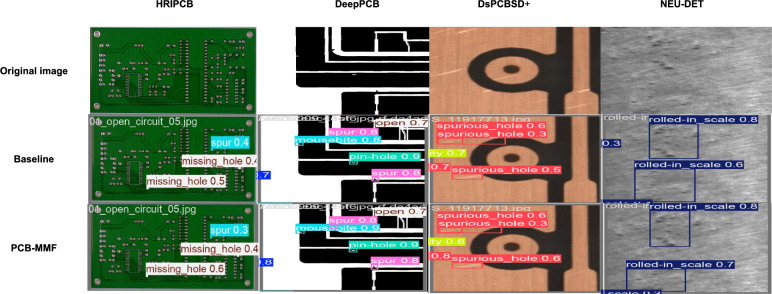
Visual comparison of detection results across four diverse industrial defect datasets. Top row: Original input images (From left to right: HRIPCB, DeepPCB, DsPCBSD+, and NEU-DET). Middle row: Detection results of the baseline YOLO11, showing occasional false positives and lower confidences. Bottom row: Detection results of the proposed PCB-MMF, demonstrating tighter localization, higher confidence scores, and effective suppression of false positives.

To intuitively verify the robust detection performance and generalization capability of the proposed model, Figure 9 provides a comprehensive visual comparison of the detection results between the baseline YOLO11 and our PCB-MMF across four diverse industrial datasets (HRIPCB, DeepPCB, DsPCBSD+, and NEU-DET).

As illustrated, the first row displays the original input images showcasing various background textures and defect morphologies. The second row shows the predictive bounding boxes generated by YOLO11, while the third row presents the results from our PCB-MMF. It can be observed that the baseline YOLO11 occasionally struggles with complex backgrounds, leading to lower confidence scores and noticeable false positives (e.g., the redundant and overlapping bounding boxes in the NEU-DET steel surface sample on the far right). In contrast, benefiting from the global-local feature fusion of MM-NET and the multi-scale attention mechanisms of MC-VAM, PCB-MMF effectively suppresses background interference. It achieves tighter bounding box localization, higher prediction confidences, and eliminates false positives. This visual evidence strongly aligns with the quantitative metrics, confirming the model’s superior sensitivity to tiny defects and its robust adaptability across distinct industrial inspection domains.

## Conclusion

The proposed PCB-MMF model demonstrates significant improvements in PCB defect detection through a hybrid architecture integrating the MM-NET backbone, the MC-VAM module, and the LGSD detection head. This design effectively alleviates challenges such as insufficient small-defect feature extraction, limited attention to complex scenarios, and excessive computational overhead. Experimental results on multiple public PCB datasets, along with further validation on the unseen NEU-DET steel surface defect dataset (achieving an mAP50 of 76.69%), indicate that our model achieves a favorable balance between detection precision and computational complexity. Compared with the YOLO11 model, PCB-MMF reduces the number of parameters by 8.9% and the computational load (FLOPs) by 12.70%, demonstrating robust architectural generalizability and broad applicability to diverse industrial inspection domains.

Despite these superior performances, a comprehensive failure-case analysis reveals certain operational boundaries. First, while Mamba layers enhance global modeling, they introduce specific computational overheads that warrant further lightweight optimization. Second, the model occasionally experiences missed detections of microscopic hairline cracks due to spatial information loss during CNN downsampling. Finally, reliance on 2D visual textures makes the model susceptible to false positives from ambiguous pseudo-defects like severe illumination artifacts or surface oxidation.

To address these limitations, our future work will focus on efficient deployment strategies, including quantization and deformable Mamba modules, as well as the integration of lightweight super-resolution enhancement for microscopic flaws. Furthermore, we plan to investigate multi-modal fusion networks incorporating 3D depth information to robustly distinguish true structural damage from surface anomalies. Finally, due to computational constraints during the extensive comparative evaluations, statistical variance analysis across multiple independent runs was not conducted. Establishing comprehensive statistical significance testing will be a standard protocol in our future algorithmic validation efforts.

## Data Availability

The datasets generated during and/or analyzed during the current study are available from the corresponding authors upon reasonable request.
